# Clinical and Parasitological Features of Patients with American Cutaneous Leishmaniasis that Did Not Respond to Treatment with Meglumine Antimoniate

**DOI:** 10.1371/journal.pntd.0004739

**Published:** 2016-05-31

**Authors:** Jairo E. Perez-Franco, Mónica L. Cruz-Barrera, Marta L. Robayo, Myriam C. Lopez, Carlos D. Daza, Angela Bedoya, Maria L. Mariño, Carlos H. Saavedra, Maria C. Echeverry

**Affiliations:** 1 Departamento de Medicina Interna, Facultad de Medicina, Universidad Nacional de Colombia, Bogotá, Colombia; 2 Unidad de infectología Hospital Militar Central, Bogotá, Colombia; 3 Departamento de Salud Publica, Facultad de Medicina Universidad Nacional de Colombia, Bogotá, Colombia; 4 Unidad de Dermatología, Hospital Militar Central, Bogotá, Colombia; 5 Facultad de Medicina Universidad Militar Nueva Granada, Bogotá, Colombia; The George Washington University School of Medicine and Health Sciences, UNITED STATES

## Abstract

**Background:**

American cutaneous leishmaniasis (ACL) is a complicated disease producing about 67.000 new cases per year. The severity of the disease depends on the parasite species; however in the vast majority of cases species confirmation is not feasible. WHO suggestion for ACL produced by *Leishmania braziliensis*, as first line treatment, are pentavalent antimonial derivatives (Glucantime or Sodium Stibogluconate) under systemic administration. According to different authors, pentavalent antimonial derivatives as treatment for ACL show a healing rate of about 75% and reasons for treatment failure are not well known.

**Methods:**

In order to characterise the clinical and parasitological features of patients with ACL that did not respond to Glucantime, a cross-sectional observational study was carried out in a cohort of 43 patients recruited in three of the Colombian Army National reference centers for complicated ACL. Clinical and paraclinical examination, and epidemiological and geographic information were recorded for each patient. Parasitological, histopathological and PCR infection confirmation were performed. Glucantime IC_50_ and *in vitro* infectivity for the isolated parasites were estimated.

**Results:**

Predominant infecting *Leishmania* species corresponds to *L*. *braziliensis* (95.4%) and 35% of the parasites isolated showed a significant decrease in *in vitro* Glucanatime susceptibility associated with previous administration of the medicament. Lesion size and *in vitro* infectivity of the parasite are negatively correlated with decline in Glucantime susceptibility (Spearman: r = (-)0,548 and r = (-)0,726; respectively).

**Conclusion:**

A negative correlation between lesion size and parasite resistance is documented. *L*. *braziliensis* was found as the main parasite species associated to lesion of patients that underwent treatment failure or relapse. The indication of a second round of treatment in therapeutic failure of ACL, produced by *L*. *braziliensis*, with pentavalent antimonial derivatives is discussable.

## Author Summary

Cutaneous leishmaniasis is tropical and subtropical worldwide vector-borne disease produced by protozoan parasites from the genus *Leishmania* that include over 20 species and produce an estimated 0.7 million to 1.3 million new cases annually. The disease features are dependent on the patient immunological stage, the parasite biology and to some extent are determined by the geographic regions in which it occurs. New World cutaneous infections known as American Cutaneous Leishmaniasis (ACL) are associated with metastatic sequelae and treatment failure. In the present study we found that in patients with ACL infected in the Colombian Amazon and Orinoco regions and not responding to Glucantime treatment, the predominant species was *L*. *braziliensis*. In addition 35% of these lesion-isolated parasites show some level of drug resistance. We also found that these resistant parasites display reduced competence when infecting the human monocytic cell line U-937 in *“in vitro”* tests and are associated with lesions of reduced size. According to our findings the indication for a second round of treatment with pentavalent antimonial derivatives in patients with ACL that did not respond to the first round of treatment is arguable.

## Introduction

Leishmaniasis is a pleiotropic syndrome caused by vector-borne protozoa of the genus *Leishmania*. Clinical classification of the disease is primarily made by its manifestation as tegumentary or visceral leishmaniasis. The clinical appearance of each case depends on the parasite species involved and the host cell-mediated immune response. Clinical stages of tegumentary disease can range from asymptomatic to localized cutaneous leishmaniasis (CL), mucocutaneous leishmaniasis (MCL), or diffuse CL (DCL) [1–3]. The disease is also classified according to the geographic regions in which it occurs. Old world leishmaniasis is present in the Middle East and Central Asia, East Africa and the Mediterranean basin [4]. New world leishmaniasis is distributed from south-central Texas through South America [5].

The new world form of CL tends to be more severe and last longer than that of the old world. In Colombia, in particular, the majority of American Cutaneous Leishmaniasis (ACL) cases are generated by species belonging to the *Leishmania (Viannia)* (L.V.) subgenus [6–8]. Species included in this last group are associated with MCL and are more prone to treatment failure than parasites from the *Leishmania (Leishmania)* (*L*.*L*.) subgenus [9–13]. Such circumstances, and the fact that species identification is not yet an option in most cases, are taken into consideration for systemic treatment to be mandatory in most ACL cases and first-line treatment is via pentavalent antimonials (sodium stibogluconate (Pentostam) or meglumine antimoniate (Glucantime)) under parenteral administration [14, 15].

The antimony treatment cure rate in ACL has been reported in meta-analyses as 76.5% (23 studies, 1,133 patients) in patients receiving 20mgSb5+/kg/day over 20 days [16], and as 75.7% in a case-controlled prospective study (119 patients) [13]. Reasons for antimony treatment failure (ATF) in ACL imply host inherent features such as age [13], drug depuration rates [17], immunological stage, drug activation and treatment adherence [18]. Natural variation in antimony susceptibility between different strains of *Leishmania spp*. has also been described and could be an important factor in antimony treatment failure in ACL [19]. However, natural parasite resistance to antimony in clinical isolates of ACL, a phenomenon in which the *in vitro* behaviour of strains does not necessarily match the clinical outcome, has been underestimated and is not well understood [20, 21].

To determine the relationship between the clinical and parasitological features of ATF in ACL patients, a serie of patients belonging to the Colombian National Army (CAN) suffering from ACL and non-responding to antimony treatment were studied. CAN counts with a strict clinical guidance for the management of ACL that includes administration of treatment under medical supervision, following up to 6 moths after treatment ends and clear clinical criteria for referral decision to a specialised level. Those criteria included patients whose do not cure with one round of Glucantime administration. Our group of study correspond with a sample of that referred patients.

Patients’ epidemiological, clinical and para-clinical characteristics were evaluated and the parasite characteristics of the strains of *Leishmania spp*. associated with the lesions, such as species, *in vitro* antimony susceptibility and *in vitro* infectivity, were analysed.

## Methods

### Study Design and Patients

This was a cross-sectional observational study. A convenience sample of 43 patients was recruited from June 2013 to March 2014. Recruitment was carried out in three national reference centres for treatment of complicated cutaneous leishmaniasis (CCL) for patients belonging to the National Colombian Army. As inclusion criteria, patients had to be male, have completed a least one round of treatment for cutaneous leishmaniasis with Glucantime 20mgSb5+/kg/day over 20 days, and experienced ATF or relapse of the primary lesion. Female patients were excluded, as were patients with incomplete or no supervised treatment. ATF was diagnosed when the patient suffered a non-healing lesion for up to six weeks after finishing the treatment. Relapse is defined as the occurrence of a new lesion inside the borders of a previous scar, within six months after healing. A non-healing lesion is defined into the CAN’s clinical guidance for the management of ACL as a lesion whose area at day 20 of treatment has not declined to 50% of the initial size or a lesion that after 6 weeks of finishing treatment has not healed. According to Olliaro et al, this ATF will correspond to an early treatment failure [22].

Patients interviews were made by following a predefined protocol containing information on demographic factors (age, birthplace, places visited in the last year) and clinical factors (clinical presentation and time of evolution of current episode of ACL, time elapsed between the appearance of symptoms and the beginning of treatment, number and site(s) of lesions, personal history of ACL, occurrence of other medical conditions, use of any prescribed or un-prescribed drug, Glucantime side effects and treatment received for the current and/or previous episodes of ACL).

The research team performed clinical evaluations and reviewed the patients’ individual medical records documented by the Colombian Army Medical Service (CAMS). Those records included information about ACL diagnosis confirmation method, drug batch, side effects and paraclinical testing during treatment. Routinely, CAMS performs paraclinical tests to evaluate complete blood count (CBC) and hepatic, pancreatic and renal function before, during, and after treatment for ACL. In patients for whom three-time points records were available this information was incorporated for analysis. Change for each enzyme was calculated simply as the ratio of the observed value to the reference upper value.

Physical examination was performed, lesion length (major axis) and width (perpendicular to the length axis) were measured and lesion size was calculated as height by width. A skin biopsy specimen from the internal border of the lesion was collected under aseptic conditions using a disposable punch (4 mm in diameter) after local anaesthetic blocking via 2% lidocaine by subcutaneous application.

### Ethics Statement

The Study was approved by the Board of Ethical Conduct of *Hospital Militar Central*-Bogotá-Colombia (HOMICE) in accordance with National (resolution 008430 of Colombian Health Ministry) and International (Declaration of Helsinki and amendments, World Medical Association, Korea 2008) guidelines. After reading and signing a written informed consent form, the patients were interviewed and examined by the research team.

### Sample Collection and Parasite Identification and Isolates

Immediately after collection, the biopsy was divided into three tissue samples. The first was placed in Senekjie medium supplemented with 1 mg/ml Biopterine and incubated at 26 ± 1°C. The second was added to a dry tube and kept at -70°C until DNA extraction, following which hsp70-PCR-RFLP designed for *Leishmania* species identification was performed as described [23]. The third was fixed in 10% buffered formalin and sent for histopathological evaluation.

### Reference Strains

Reference strains *of L*.*(V)*. *braziliensis* (MHOM/BR/75/M2903) and *L*.*(V)*. *guyanensis* (MHOM/GF/79/LEM85) were included as a control for infectivity and Glucantime susceptibility assays. Promastigotes of reference strains were kept on Schneider (Gibco, UK) supplemented with 10% heat-inactivated foetal calf serum (FCS), penicillin (50 units/mL) and streptomycin (50 μg/mL) and incubated at 26 ± 1°C.

### Infectivity of Parasites to the U-937 Cell Line

U-937 cells were seeded 4 x 10^5^ per well in a 6-well plate containing a sterile slide in RPMI 10% FCS medium. To induce cell differentiation, 12-myristate, phorbol 13-acetate (PMA), was added at 100 ng/ml. Cells were differentiated to adherent non-dividing cells by incubation for 5 days at 37°C with an atmosphere of 5% CO_2_. Differentiated cells were infected and infections were kept for 48 hours at 34°C. Percentage of infection was calculated by microscopic visualisation of Giemsa-stained slides and corresponded to the number of infected macrophages from 100 macrophages counted. Reading was carried out in triplicate and performed by two independent readers.

### *In vitro* sensitivity tests

U937 *Leishmania (V) braziliensis or guyanensis* infected cells were maintained in RPMI 10% FCS medium at 34°C in a 5% CO_2_ incubator with high relative humidity. After 48 hours of infection, the culture medium was replaced by medium containing Glucantime at concentrations of 0, 28, 32, 64, 128 and 256 μg/ml. Each treatment point was seeded in triplicate. Chambers were washed with RPMI on day 2 and a new Glucantime-RPMI-FCS preparation was then added to the cells. After three days of treatment, medium was removed before the slides were washed, dried, and stained with Giemsa solution. The ratio of *Leishmania* intracellular parasites to U-937 cells was determined for each Glucantime concentration as described in the previous section. Two independent readers performed readings in triplicate. The 50% effective concentration (IC_50_) corresponded to the concentration of Glucantime that reduced the survival of *Leishmania* parasites by half and was determined using the CONFINT function of the R programme.

### Statistical Analysis

Probit curves were generated to analyse the percentage of parasites’ survival inhibition at each drug concentration. In cases with significant variation in the replicas, two to three curves were obtained for each group of data. In these cases, the IC_50_ intervals were considered for the whole spectrum of data distribution.

Stata version 11 (StataCorp, 2005, College Station, TX) was used for analyses. Interquartile range (IQR) was used to describe most demographic and clinical variables rather than medians as the study populations had a non-normal distribution, requiring non-parametric analysis.

In order to determine whether there was any correspondence of parasitological features of isolates (e.g. resistance grade, *in vitro* infectivity) with the clinical features of the lesion (e.g. lesion type, number and size), correlation tests (Spearman’s rank and Pearson's Chi Squared test) were performed.

## Results

With the aim of illustrating relevant clinical and parasitological features among ACL patients with ATF, and their potential relationship, we evaluated a sample of 43 men who had been referred to a specialist centre due to an ACL event considered to be an ATF (n = 35) or a relapse (n = 8). Individuals voluntarily agreed to participate in the study. A summary of patient profiles is presented in Table 1, showing that the group was made up of healthy young men. Coexistence of ACL with infectious and non-infectious diseases was investigated, and found not to be significant. Patients were infected in Colombian Amazonian and Orinoco regions. Detailed clinical description of patients is included in supporting information (S1 and S2 Tables).

**Table 1:** Clinical features of patients’ cohort.

*Variable*	Cohort Distribution (quartiles)				
	Q1	Q2	Q3	Smallest	Largest
Age	23	25	27	21	39
Height (cm)	163	169	175	157	184
Weight (kg)	63	67.5	78	55	115
BMI^1^	22.5	24.4	26.5	18.9	34.7
Evolution time^2^	16	32	48	10	192
Time for treatment^3^ (weeks)	5	8	12	3	60
Time from end of treatment ^4^ (weeks)	6.5	23	31	0**	113
Lesion area (cm^2^)	4.4	7.5	12	1.68	190*
Haemoglobin	15.6	16	16.9	13.7	17.6
White blood cells counting	6,070	7,500	8,700	4,000	11,310
Platelets	247,500	290,500	323,500	200,000	393,000
Lymphocyte (%)	25	33	43	12	58
Neutrophil (%)	46	50	60	23	72
Eosinophil (%)	2.8	4.5	6.6	1.2	12

^1^ Body mass index

^2^ Time (in weeks) between date of diagnosis confirmation and date of patient recruitment for the study

^3^ Elapsed time between the onset of symptoms and initiation of treatment with Glucantime

^4^ Time (in weeks) between date of treatment end and date of patient recruitment for the study

*Highest value corresponds to a lesion of 20 weeks of evolution on the right leg of a patient who presented multiple lesions and a BMI of 30.47

** Corresponding to a patient that was just ending the treatment without response during the time of the study recruitment

51% of the patients suffered a unique lesion, 32% presented 2–4 lesions and 17% suffered from 5–16 lesions. Most patients had lesions on their upper limbs (39.5%), multiple lesions differentially located (30.2%) or lesions located mainly on the face and neck (16.3%). Less frequently, lesions were found on legs (11.6%) and the thoracic-abdominal region (2.3%).

Predominant lesion types corresponded with ulcerative lesions as well as nodular, plaques and papular-type of injuries. Lesion size median value was 14.74cm^2^ with minimal value of 1.68cm^2^ and maximal of 190 cm^2^.

All patients had to have received a least one complete round of Glucantime (20mgSb5+/kg/day over 20 days). A number of patients had had two (13.9%) or three (4,7%) complete cycles.

The data presented in this study concerning side effects and blood tests correspond with the information regarding the last Glucantime round received by the patients, and to which the patients did not respond. The most common side effects described after therapy with antimony salts were: arthralgia (41.8%), headache (30.2%), fever (30.2%), myalgia (25.5%), anorexia (11.6%), nausea (9.3%), diarrhoea (9.3%), and other symptoms such as chest pain, tachycardia and facial swelling (13.95%).

Blood testing of antimony treatment side effects on renal function showed no alteration in blood urea nitrogen (BUN), nor in creatinine levels. However Aspartate Aminotransferase (AST) and Alanine Aminotransferase (ALT) were raised during treatment in some patients to 2.6 (n = 4) and 5.7 (n = 8) times the upper reference limit, respectively, with normal values regained after the end of treatment. Values went out of the normal range 30 days after patients finished treatment, with values rising to 2.3 (n = 3) and 5 (n = 3) times the upper reference limit for AST and ALT, respectively (S1A and S1B Fig).

Results for serum amylase differed because of the presence of a group of patients with basal levels out of range before the beginning of treatment. These patients were 3.5 times (n = 12) above the upper reference limit during treatment but came back to normal values after antimony treatment was suspended (S1C Fig). The increased amylase basal level was related to patients who had recently finished a complete Glucantime round.

Parasitological diagnosis was assessed at the beginning of the study by microscopic examination of Giemsa-stained smears (51.2%) or histopathological analysis (46.5%). Only one patient, accounting for 2.3% of the samples, needed molecular confirmation of *Leishmania* infection.

As described in the methods section, a biopsy was taken from each patient in order to perform PCR-RFLP species identification, isolate the parasitic strain and enable histopathological analysis. In 20% of patients whose initial diagnosis was made by biopsy examination, the histopathological report of the new biopsy was negative for ACL (non-conclusive or non-compatible). However, in 50% of histopathologically negative samples, culturing allowed parasite isolation and parasite DNA was amplified from 100% of these patients.

In 95.4% of the total number of cases, *L*. *(V) braziliensis* was associated with the lesions and 2.3% were due to *L*. *(V) guyanensis*. Of the total number of patient samples, only one (2.3%), was negative for nested-PCR parasite DNA amplification, despite being reported as positive by Giemsa staining.

In 53.5% of the samples, it was possible to isolate the parasite from patients’ biopsies, although one strain was lost after isolation due to cell culture contamination. Isolation success did not depend on variables such as lesion evolution time, time elapsed between sample acquisition and end of last treatment, lesion size, lesion location or number of lesions (S2 Table) (chi-square, p>0.05).

**Fig 1:**
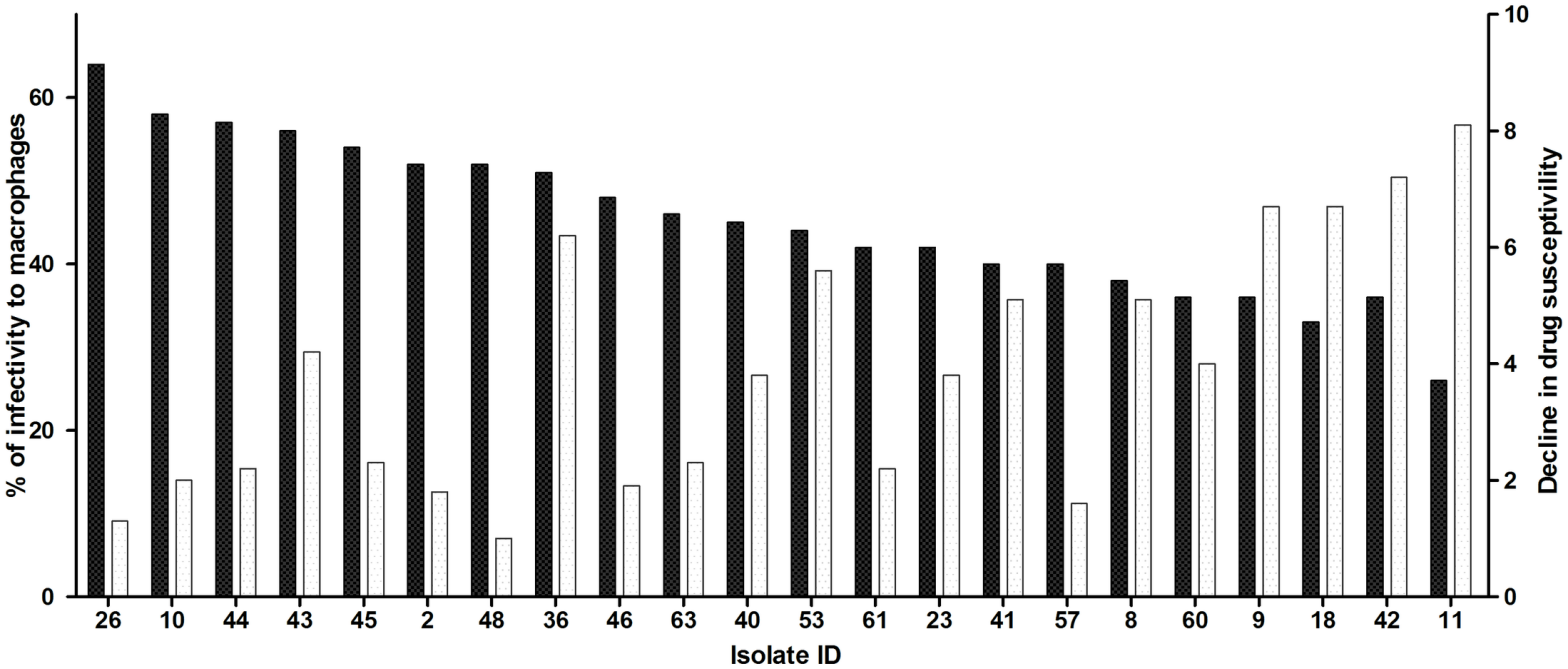
In vitro behaviour of patient’s isolates. The graph presents the *in vitro* Infectivity of *Leishmania spp*. clinical isolates to macrophages (dark bars) and fold chance in Susceptibility to Glucantime (clear bars). Percentage of infectivity corresponds with the number of infected macrophages from 100 macrophages counted. Change in sensitivity to Glucantime of Patient Clinical isolates was calculated as the ratio between the average of inhibitory dose 50 (IC_50_) of each isolate and the average of IC_50_ of the reference strain (S3 Table).

Using isolated strains, intracellular forms were obtained by infecting U-937 monocyte cell lines. The susceptibility of intracellular amastigotes to Glucantime was expressed as fold-change, calculated as the ratio of the average of isolate IC_50_ value to the average reference strain IC_50_ value. In 45% of the isolates, susceptibility fold-change was less than 3-fold due to the broadness of IC_50_ ranges (S2 Fig). This group was considered to be susceptible as reference strains. In 20% of the clinical isolates, susceptibility to the drug decreased 3.01–4.9-fold in comparison to the reference strain, and a decline in susceptibility of 5–8-fold was observed in 35% of the isolates (Fig 1- clear bars). Henceforth, this loss in susceptibility will be presented as resistance grade.

The *in vitro* infectivity of the clinical isolates was assessed through calculation of the percentage of infectivity of a human differentiated macrophage cell line (U-937). In reference strains *L*.*(V)*. *braziliensis* (MHOM/BR/75/M2903) and *L*.*(V)*. *guyanenesis* (MHOM/GF/79/LEM85), infectivity was 60% and 45%, respectively. In the parasites isolated from patients the infectivity ranged from 26–65% (Fig 1- dark bars).

**Fig 2:**
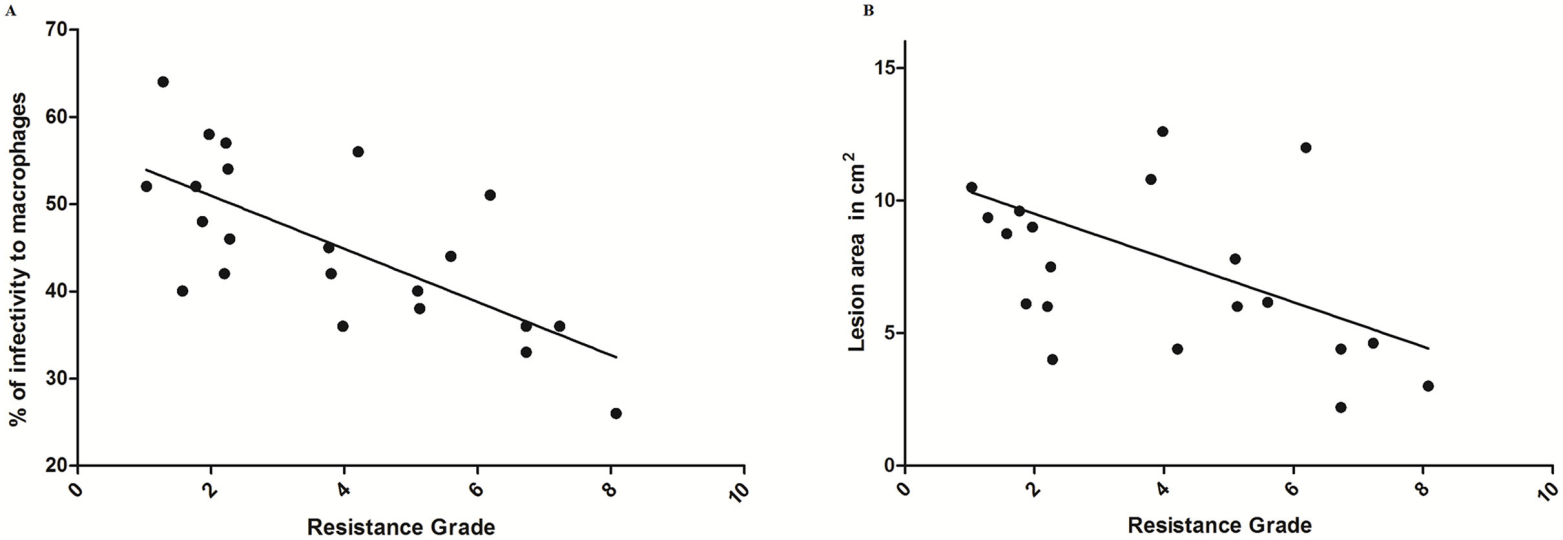
Increase in resistance grade is correlated with the loss of *in vitro* parasite infectivity and decreased cutaneous lesion size. Behaviour of the data where correlation was statistically certain are shown. **A.** Graph presenting the distribution of the percentage of *in vitro* infectivity as a function of their corresponding resistance grade for each isolate (Spearman r -0,7, p = 0.0006,95% confidence interval -0.86 to -0.34). **B.** Graph presenting the lesion size distribution as a function of their corresponding isolates’ resistance grade (Spearman r -0,5, p = 0.019, 95% confidence interval -0.775 to -0.0806). Graph and analyses were made with GraphPad Software.

When the resistance grade of the isolates was considered as the independent variable, there was a negative correlation with the *in vitro* infectivity percentage (r = -0.726, 95% CI (-0.86 to -0.34) p = 0.0006, Spearman) (Fig 2A. The resistance grade of the isolates also showed a negative correlation with the lesions’ size (r = -0.548, 95% CI (-0.775 to -0.0806), p = 0.019, Spearman) (Fig 2B). However, there was no association between *in vitro* infectivity and the size of the lesions (r = 0.433, 95% CI (-0.0416 to 0.748), p = 0.0644, Spearman). Other variables analysed showed no correlation (data no shown).

## Discussion

Pentavalent antimonies are the first choice treatment for ACL with a healing rate of about 75%. However, treatment response is highly dependent on the parasite species and host immune background. In cases of ATF in ACL for the first cycle of AM, as in cases of relapse, The World Health Organization Expert Committee and many national clinical guidelines in Latin American countries recommend a second AM cycle of treatment [4, 13, 16, 24, 25].

In our series of patients, 18% underwent treatment with a second or third cycle of AM without lesion resolution. This finding is in accordance with reported healing rates for the third AM cycle decreasing by 50% [16, 26]. Hepatic, renal and pancreatic functions evaluated through paraclinical tests imply that drug tolerance is not a problem associated with ATF or relapse in this patient series. Furthermore, 25.5% of the patients had also received second line drugs such as Pentamidine and Miltefosine without lesion healing, suggesting that any treatment for ACL requires some ability of the patient to achieve parasite clearance. This idea is supported by the fact that, in the present study, parasite DNA was found in 98% of cases, and parasites were easily isolated in 53.5%.

The fact that the predominant species was *L*.*(V)*. *braziliensis* is in agreement with other studies that have shown this species as often being refractory to AT, especially in the Amazon-Orinoco regions, where most of our patients come from [13, 27]. This finding could be useful at the clinical level in considering ATF risk when parasite species determination is not possible.

The finding that 35% of the isolates presented a considerable decrease in Glucantime susceptibility could only partially explain ATF. The two isolates that presented the highest resistance grade (Fig 1 isolates 11 and 42) came from patients that had had previous ACL episodes that were treated with AM (three cycles for isolate11 and two for isolate 42) (S1 Table). Therefore, these parasites could have been progressively selected *in vivo* in healed scars as hidden viable parasites [28, 29], as just one cycle of Glucantime is enough to increasing the IC_50_ for parasites associated with lesions when evaluated before and after treatment [20, 30]. In addition, there is evidence that an antimonial host transporter localized at the cell and phago-lysosomal membranes of macrophages undergoes differential expression levels in infected cells treated with antimony, thus, regulating the antimony concentrations to which amastigotes are exposed [31].

The remaining isolates with high resistance grade (Fig 1 Isolates 8,9,18,36,41,53) corresponded to patients that were in their first ACL episode and had received just one round of AM (S2 Table). In these cases, primary resistance could be considered, implying the anthroponotic transmission of *Leishmania braziliensis* resistant strains [20, 29].

Epidemiological and clinical variables regarding disease characteristics, patients’ background and Glucantime tolerance did not show any remarkable features in patients under ATF, and had similar frequency and distribution as in other series of military patients with ACL who responded to AT and patient series’ from different American countries [9, 13, 32–35].

In the present study, an inverse relationship between *in vitro* Glucantime susceptibility and *in vitro* infectivity was demonstrated (Fig 2A), suggesting that isolates with reduced susceptibility undergo a reduction in fitness. This last finding could be the result of a decrease in cell invasion ability or a reduced replication rate.

The most notable finding was that lesion size showed no association and was not dependent on disease duration. However there was an inverse relationship between isolate resistance grades and lesion size (Fig 2B). Case-control studies had previously shown that presence of “concomitant-distant” lesions, duration of the disease and total lesion area (calculated as areas of the three largest lesions) are significantly associated with ATF In ACL [11] In our series, most of patients presented a unique lesion (51%) and in this subgroup no association between lesion area and duration of the disease was found or in the group with more than 2 lesions. These apparently controversial results can be explained because in the current study the number of patients suffering 3 o more lesions was scarce (n = 16) and because the analysis did not consider cumulative lesion areas when more than one lesion was present.

Several authors agree that treatment failure is an outcome with multiple origins, which include parasite resistance in a percentage of cases [9, 13, 18, 20, 29, 36]. In our series of patients, the corresponding 35% displayed lesions of reduced size independently of disease duration. This finding implies reinterpretation of previous reports that initially suggested -in bivariate analysis- an increased risk of small lesions for ATF; the association was lost -in the multivariate model- because of the interaction with duration of disease [13]. In that study the Antimony IC_50_ for parasites associated to the lesions was not determined, and given the multifactorial origin of ATF, it is plausible that in such a big cohort a fraction of patients could be infected with parasites with decreased drug sensitivity. According to our findings in that case, if patients are infected with less sensitive *L*. *braziliensis*, the initial observed increased risk of small lesions for ATF would be maintained independently of the duration of the disease. This finding could be tested using a case-control study design involving patients who do respond to antimony treatment and assessing IC_50_ for lesion-associated parasites.

Nevertheless, there was no correlation between lesion size and *in vitro* infectivity, meaning that the lesion progression mechanism in LCA associated to *L braziliensis* is not highly dependent on parasite infectivity and could be more associated to the host immunity as suggested for recent studies [37–39].

In conclusion, the fact that 55% of isolates from patients under ATF showed a decrease in Glucantime susceptibility, and that most resistant isolates came from patients who had received two or more rounds of AM for ACL treatment, suggests that the practice of recommending ACL patients for a second round of AM treatment to deal with an ATF or relapse episode should be reviewed. Moreover, the finding that 95.5% of lesions in patients with ATF were associated to *L*. *braziliensis* in a region where other species have also been reported [6–8] is in agreement with findings from others [9,12,13], reasserting that LCA produced by *L*. *braziliensis* is generally more difficult to cure and concurs with a higher relapse rate. This consideration implies that the therapeutic approach in ACL should be based on identification of the parasite species, and a combination treatment for LCA associated to *L*. *braziliensis* could be contemplated as a first line of treatment.

## Supporting Information

S1 Table Summary of Clinical Features of Patients without Parasite Isolation (PDF)

S2 Table Main clinical features of patients with parasitic isolation. (PDF)

S3 Table Average Value of IC_50_ in (μg/ml) calculated for reference strains and patients’ isolates. Data were assembled according to the readings describing percentage of infection of control isolates, (*L (V)*. *braziliensis* (MHOM/BR/75/M2903) and *L (V)*. *guyanensis* (MHOM/GF/79/LEM85) to U-937 cells. To account for biases during microscopic readings each reader was provided with blind slides and the data compared with the readings performed by the same reader on the control slides. Statistical replicas are presented in the table and correspond to those cases where variation between data did not allowed a unique curve. In those cases three probit curves were generated. NV cells are data groups where only one curve was generated, meaning there was no variation between statistical replicas.
(PDF)

S1 Fig Hepatic and Pancreatic paraclinical follow-up during Gucantime treatment for ACL. Serological levels of Aspartate Aminotransferase (AST) (**A)**; Alanine Aminotransferase (ALT) **(B)**; and Amylase **(C)** were assessed before, during and 30 days post treatment. Change for each enzyme was calculated as the ratio of the observed value (Individual) to the reference upper value.
(TIF)

S2 Fig Probit curves obtained for reference strains and patients’ isolates. IC_50_ intervals are presented.
(PDF)
