# Supplementary material for: Clinical and Parasitological Features of Patients with American Cutaneous Leishmaniasis that Did Not Respond to Treatment with Meglumine Antimoniate
Source: PLoS Negl Trop Dis. 2016 May 31;10(5):e0004739. doi: 10.1371/journal.pntd.0004739 (PMC4887049; doi:10.1371/journal.pntd.0004739)
Supplement: S1 Table — (PDF) [file pntd.0004739.s001.pdf]

Patient ID	Leishmania species	Topology	# of lesions	Lesion size(cm^2)[i]	Evolution (weeks)	Time of the Last Treatment[ii]	Geographic procedence[iii]	# of AM-Treatments[iv]	# of Non - AM-treatment[v]	Treatment outcome	Previous Episodes	Previous AM treatment[vi]
1	*L.braziliensis*	UP[1]	1	13,3	16	6	1	1	0	Failure	0	0
3	*L.braziliensis*	UP	1	7,5	57	11	1	1	0	Failure	1	1
4	*L.braziliensis*	UP	1	1,68	20	9	4	1	0	Failure	0	0
5	*L.braziliensis*	face&neck	3	8,99	40	21	4	1	0	Failure	0	0
6	*L.braziliensis*	face&neck	1	13,2	11	5	4	1	0	Failure	0	0
12	*L.braziliensis*	ML[2]	3	5	28	5	4	1	0	Failure	0	0
13	*L.braziliensis*	Legs	6	6	14	4	4	1	0	Failure	0	0
14	*L.braziliensis*	Thoracic-abdominal	3	1,8	28	25	4	1	0	Failure	0	0
15	*L.braziliensis*	UP	1	15	16	25	4	1	0	Failure	0	0
16	*L.braziliensis*	ML	5	2,25	32	25	4	1	0	Failure	0	0
17	*L.braziliensis*	ML	1	2,55	16	5	NI[3]	1	0	Failure	1	1
19	NI[3]	Legs	2	26,5	36	29	4	2	0	Failure	0	0
20	*L.braziliensis*	Legs	3	2,56	20	5	4	1	0	Failure	0	0
21	*L.braziliensis*	ML	3	10	16	5	4	1	0	Failure	0	0
28	*L.braziliensis*	UP	1	95	192	112	1	3	1	Relapse	0	0
29	*L.braziliensis*	ML	3	2,5	41	33	4	2	0	Failure	0	0
34	*L.braziliensis*	Legs	1	8,75	32	9	4	1	0	Failure	0	0
35	*L.braziliensis*	ML	4	2,25	16	9	4	1	0	Failure	0	0
47	*L.braziliensis*	ML	10	6	128	113	1	3	1	Failure	0	0
59	*L.braziliensis*	face&neck	1	22,5	96	29	4	1	0	Failure	0	0
62	*L.braziliensis*	UP	2	24	36	NI	4	2	4	Relapse	0	0

[1]UP, Upper Limbs
[2] ML, Multiple lesions located in different topology
[3] NI, No information

[i] The biggest lesion in cases with multiple lesions
[ii] Weeks elapsed between the end of treatment and the date of patient recruitment
[iii] Internal code assigned for the study.
[iv] Number of treatments received for this lesion with AM
[v] Treatments received for this lesion with drugs different from AM
[vi] Number of treatments received for previous ALC. episodies
