# Supplementary material for: Clinical and Parasitological Features of Patients with American Cutaneous Leishmaniasis that Did Not Respond to Treatment with Meglumine Antimoniate
Source: PLoS Negl Trop Dis. 2016 May 31;10(5):e0004739. doi: 10.1371/journal.pntd.0004739 (PMC4887049; doi:10.1371/journal.pntd.0004739)
Supplement: S2 Table — (PDF) [file pntd.0004739.s002.pdf]

Patiente (Isolate) ID	Leishmania species	Topology	# of lesions	Lesion size(cm^2)[i]	Evolution (weeks)	Time of the Last Treatment[ii]	Geographic procedence[iii]	# of AM-Treatments[iv]	# of Non -AM-treatment [v]	Treatment outcome	Previous Episodes	Previous AM treatment[vi]
2	*L.braziliensis*	face&neck	1	9,6	36	26	4	1	1	Failure	0	0
8	*L.braziliensis*	ML[vii]	13	6	12	NI[viii]	4	1	0	Failure	0	0
9	*L.braziliensis*	ML	2	2,25	12	0	4	1	0	Failure	0	0
10	*L.braziliensis*	UP[ix]	1	9	12	5	4	1	0	Failure	0	0
11	*L.braziliensis*	Legs	1	3	10	7	4	1	1	Failure	1	3
18	*L.braziliensis*	UP	1	4,4	36	30	4	1	0	Failure	0	0
23	*L.braziliensis*	UP	1	10,8	16	8	1	1	0	Relapse	0	0
26	*L.braziliensis*	UP	1	9,35	44	41	4	1	1	Failure	0	0
44	*L.braziliensis*	UP	1	22	56	53	4	1	2	Failure	0	0
36	*L.braziliensis*	UP	1	12	44	41	4	1	1	Failure	0	0
40	*L.braziliensis*	ML	9	190	20	4	1	1	0	Failure	0	0
41	*L.braziliensis*	UP	1	7,8	20	8	1	1	0	Failure	0	0
42	*L.braziliensis*	UP	1	4,62	40	37	1	1	0	Relapse	1	1
43	*L.guyanensis*	face&neck	1	4,4	40	37	5	2	0	Relapse	0	0
45	*L.braziliensis*	ML	4	7,5	64	49	4	1	0	Failure	0	0
46	*L.braziliensis*	UP	1	6,1	124	113	4	1	2	Relapse	0	0
48	*L.braziliensis*	ML	16	10,5	23	16	4	1	0	Failure	0	0
60	*L.braziliensis*	ML	5	6	52	45	4	1	2	Failure	0	0
53	*L.braziliensis*	UP	0	6,16	26	15	1	1	1	Failure	0	0
57	*L.braziliensis*	face&neck	1	8,75	48	45	1	2	0	Failure	0	0
61	*L.braziliensis*	UP	3	12,6	60	NI	5	1	0	Relapse	0	0
63	*L.braziliensis*	face&neck	2	4	76	NI	4	2	0	Relapse	0	0

[i] The biggest lesion in cases with multiple lesions
[ii] Weeks elapsed between the end of treatment and the parasitic isolation.
[iii] Internal code assigned for the study.
[iv] Number of treatments received for this lesion with AM
[v] Treatments received for this lesion with drugs different from AM
[vi] Number of treatments received for previous LC. episodies
[vii]ML, multiple lesion located with different topology
[viii]NI, no information
[ix] UP, upper Limbs
