# Supplementary material for: Clinical and Parasitological Features of Patients with American Cutaneous Leishmaniasis that Did Not Respond to Treatment with Meglumine Antimoniate
Source: PLoS Negl Trop Dis. 2016 May 31;10(5):e0004739. doi: 10.1371/journal.pntd.0004739 (PMC4887049; doi:10.1371/journal.pntd.0004739)
Supplement: S3 Table — Data were assembled according to the readings describing percentage of infection of control isolates, (L (V). braziliensis (MHOM/BR/75/M2903) and L (V). guyanensis (MHOM/GF/79/LEM85) to U-937 cells. To account for biases during microscopic readings each reader was provided with blind slides and the data compared with the readings performed by the same reader on the control slides. Statistical replicas are presented in the table and correspond to those cases where variation between data did not allowed a unique curve. In those cases three probit curves were generated. NV cells are data groups where only one curve was generated, meaning there was no variation between statistical replicas. (PDF) [file pntd.0004739.s003.pdf]

		Average Value of IC_{50} in (μg/ml)			
		Replica 1	Replica 2	Replica 3	Average
Group 1 *L. (V). braziliensis* (MHOM/BR/75/M2903)	Control	5	26	47	26
	Patient 8	96	132	171	133
	Patient 9	127	173	222	174
	Patient 46	147	109	70	109
	Patient 53	153	145	137	145
	Patient 10	41	52	59	51
	Patient 18	116	173	233	174
	Patient 11	209	NV	NV	209
	Patient 2	83	103	124	103
	Patient 57	26	41	54	41
	Patient 40	109	98	85	97
	Patient 36	160	NV	NV	160
	Patient 23	132	98	65	98
	Patient 60	103	NV	NV	103
	Patient 41	142	132	122	132
	Patient 42	186	NV	NV	186
Group 2 *L. (V). braziliensis* (MHOM/BR/75/M2903)	Control	65	54	47	55
	Patient 44	111	122	134	122
	Patient 48	57	NV	NV	57
	Patient 45	124	NV	NV	124
	Patient 26	80	70	62	71
	Patient 61	160	119	78	119
	Patient 63	127	NV	NV	127
Group 3 *L. (V). guyanensis* (MHOM/GF/79/LEM85)	Control	54	NV	NV	54
	Patient 43	246	228	209	228

Data were assembled according to the readings describing percentage of infection of control isolates, (*L (V). braziliensis* (MHOM/BR/75/M2903) and *L (V). guyanensis* (MHOM/GF/79/LEM85) to U-937 cells. To account for biases during microscopic readings each reader was provided with blind slides and the data compared with the readings performed by the same reader on the control slides.

Statistical replicas are presented in the table and correspond to those cases where variation between data did not allowed a unique curve. In those cases three probit curves were generated. NV cells are data groups where only one curve was generated, meaning there was no variation between statistical replicas.
