# Supplementary figures and images for: Clinical and Parasitological Features of Patients with American Cutaneous Leishmaniasis that Did Not Respond to Treatment with Meglumine Antimoniate
Source: PLoS Negl Trop Dis. 2016 May 31;10(5):e0004739. doi: 10.1371/journal.pntd.0004739 (PMC4887049; doi:10.1371/journal.pntd.0004739)

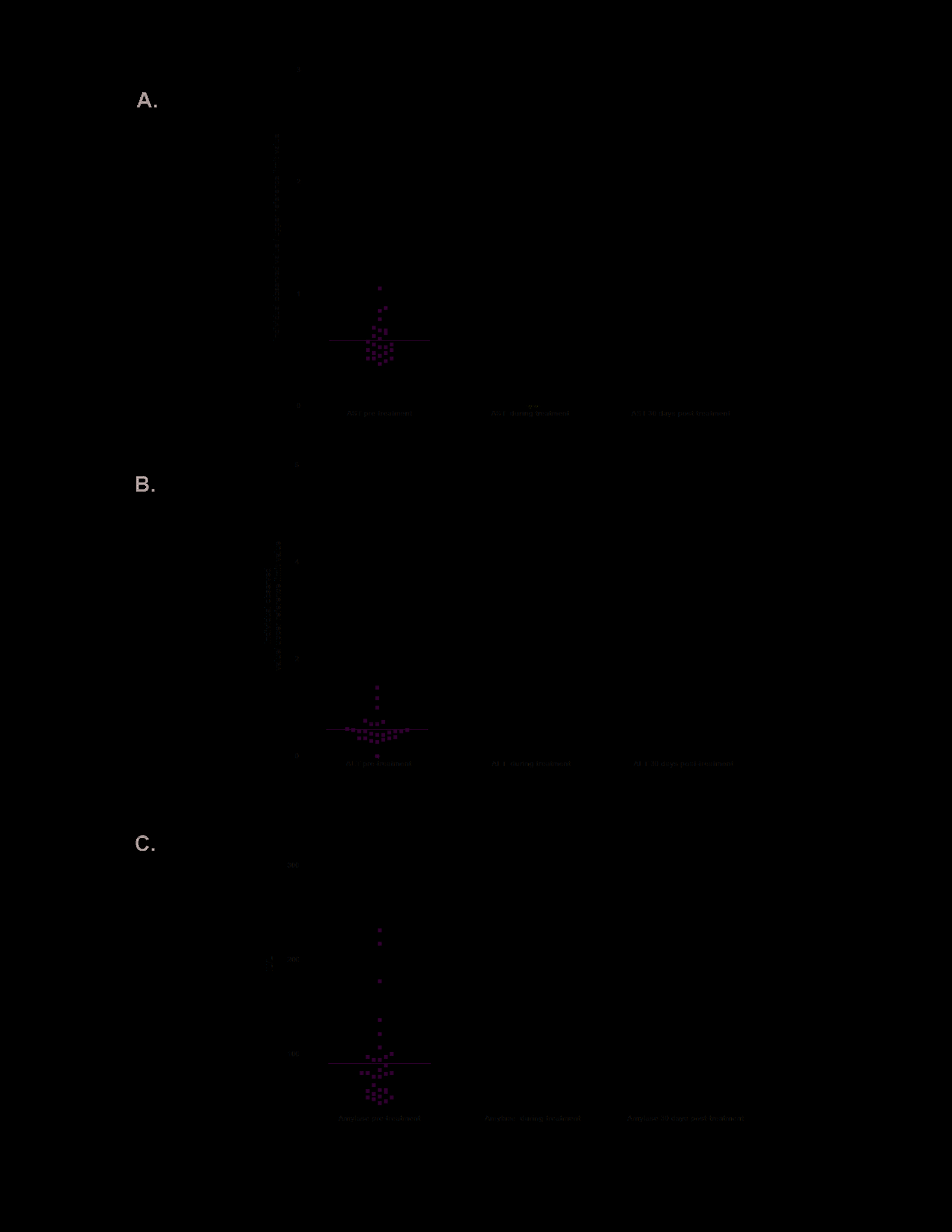

Supplement: S1 Fig — Serological levels of Aspartate Aminotransferase (AST) (A); Alanine Aminotransferase (ALT) (B); and Amylase (C) were assessed before, during and 30 days post treatment. Change for each enzyme was calculated as the ratio of the observed value (Individual) to the reference upper value. (TIF) [file pntd.0004739.s004.tif]
