# Supplementary material for: Clinical and Parasitological Features of Patients with American Cutaneous Leishmaniasis that Did Not Respond to Treatment with Meglumine Antimoniate
Source: PLoS Negl Trop Dis. 2016 May 31;10(5):e0004739. doi: 10.1371/journal.pntd.0004739 (PMC4887049; doi:10.1371/journal.pntd.0004739)
Supplement: S2 Fig — IC50 intervals are presented. (PDF) [file pntd.0004739.s005.pdf]

Control Group 1

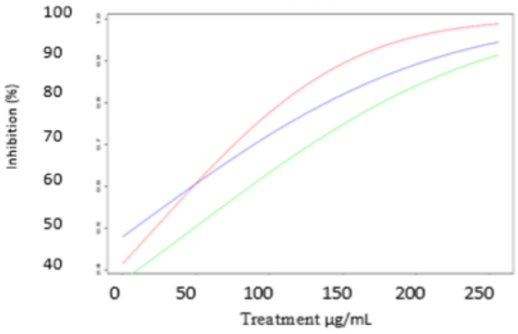


```
Coefficients:
              Estimate Std. Error z value Pr(>|z|)
(Intercept)  0.1142904  0.0286129   3.994 6.49e-05 ***
Treatment    0.0073122  0.0001544  47.344  < 2e-16 ***
Replication -0.1507419  0.0124034 -12.153  < 2e-16 ***
```

	DL 50	LI	LS
Replication	5.171	-5.818	16.666
Replication	25.858	10.804	41.593
Replication	46.545	27.428	66.519

Isolate Patient 8

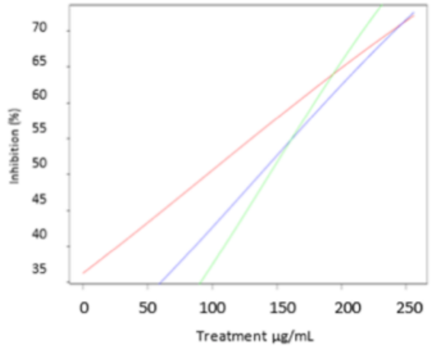


```
Coefficients:
              Estimate Std. Error z value Pr(>|z|)
(Intercept) -0.2800590  0.0434581  -6.444 1.16e-10 ***
Treatment    0.0049738  0.0001916  25.962  < 2e-16 ***
Replication -0.1908983  0.0207795  -9.187  < 2e-16 ***
```

	DL 50	LI	LS
Replication	95.677	64.495	129.801
Replication	131.878	92.565	180.172
Replication	170.667	120.634	230.543

Isolate Patient 9

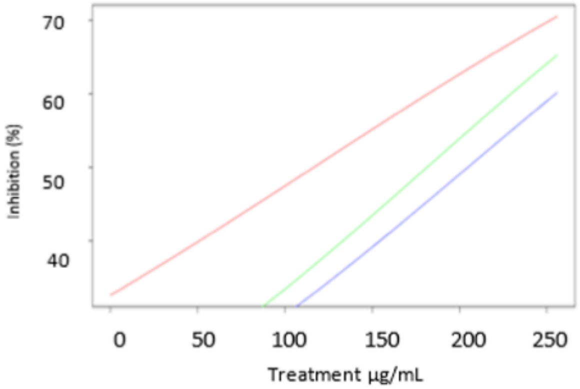


```
Coefficients:
              Estimate Std. Error z value Pr(>|z|)
(Intercept) -0.3550922  0.0408054  -8.702   <2e-16 ***
Treatment    0.0045397  0.0001803  25.172   <2e-16 ***
Replication -0.2167800  0.0195758 -11.074   <2e-16 ***
```

	DL 50	LI	LS
Replication	126.707	92.748	164.821
Replication	173.252	129.262	225.715
Replication	222.384	165.775	286.608

Isolate Patient 46

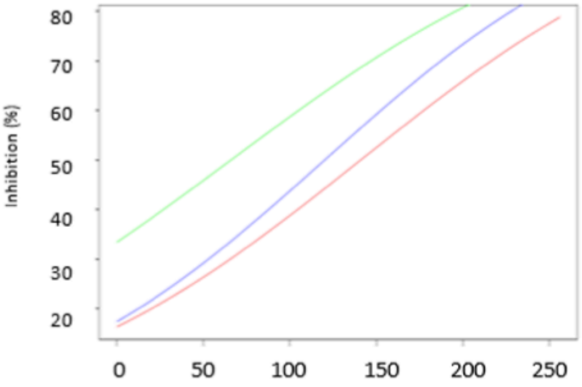


```
Coefficients:
              Estimate Std. Error z value Pr(>|z|)
(Intercept) -1.2926925  0.0368649  -35.07   <2e-16 ***
Treatment    0.0069107  0.0001505   45.92   <2e-16 ***
Replication  0.2699006  0.0140553   19.20   <2e-16 ***
```

	DL 50	LI	LS
Replication	147.394	169.454	128.269
Replication	108.606	86.959	132.849
Replication	69.818	45.650	96.245

Isolate Patient 53

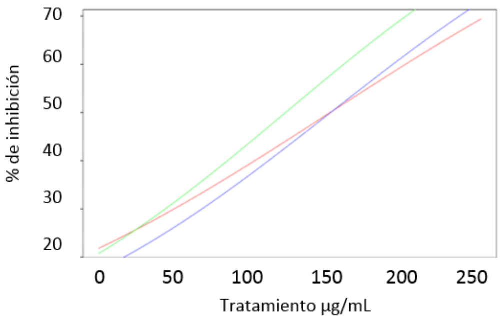


```
Coefficients:
              Estimate Std. Error z value Pr(>|z|)
(Intercept) -0.9435694  0.0412931 -22.851  < 2e-16 ***
tratamiento  0.0058480  0.0001648  35.488  < 2e-16 ***
bloques      0.0463504  0.0179795   2.578  0.00994 **
```

	DL 50	LI	LS
Bloque 1	152.565	126.583	183.415
Bloque 2	144.808	113.379	181.384
Bloque 3	137.050	100.175	179.352

Isolate Patient 10

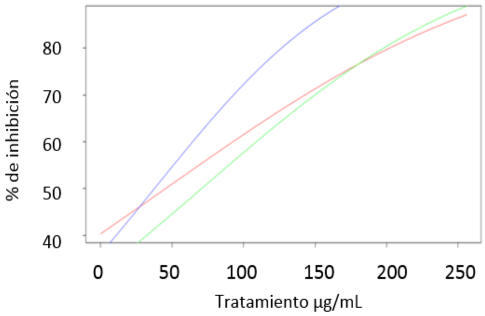


```
Coefficients:
              Estimate Std. Error z value Pr(>|z|)
(Intercept) -0.2244816  0.0343326  -6.538 6.22e-11 ***
tratamiento  0.0066629  0.0001775  37.540  < 2e-16 ***
bloques     -0.0574359  0.0154318  -3.722 0.000198 ***
```

	DL 50	LI	LS
Bloque 1	41.373	26.351	59.948
Bloque 2	51.717	30.246	73.789
Bloque 3	59.474	87.630	34.141

Isolate Patient 18

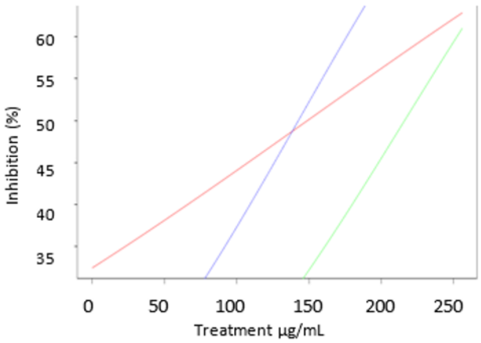


```
Coefficients:
              Estimate Std. Error z value Pr(>|z|)
(Intercept) -0.3154095  0.0797853  -3.953 7.71e-05 ***
Treatment    0.0055721  0.0003429  16.248  < 2e-16 ***
Replication -0.3267237  0.0381657  -8.561  < 2e-16 ***
```

	DL 50	LI	LS
Replication	116.363	65.944	178.077
Replication	173.252	106.347	259.965
Replication	232.727	146.749	341.853

Isolate Patient 11

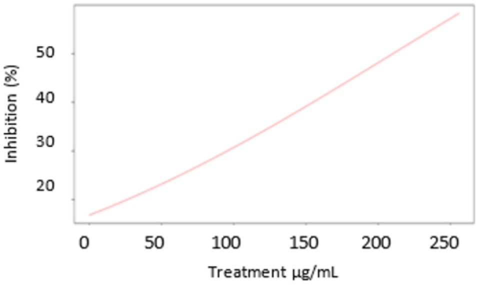


```
Coefficients:
              Estimate Std. Error z value Pr(>|z|)
(Intercept) -1.0200130  0.0871962  -11.70   <2e-16 ***
Treatment    0.0045620  0.0003544   12.87   <2e-16 ***
Replication  0.0312841  0.0381684    0.82    0.412
```

	DL 50	LI	LS
Replication	209.454	165.111	272.061

Isolate Patient 2

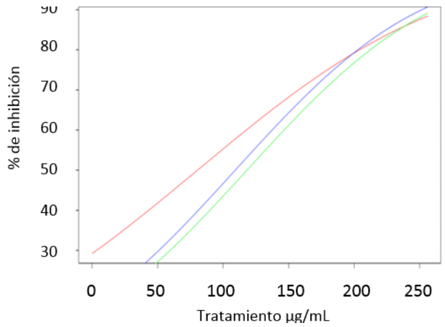


```
Coefficients:
             Estimate Std. Error z value Pr(>|z|)
(Intercept) -0.5282988  0.0503333 -10.496  < 2e-16 ***
tratamiento  0.0083127  0.0002304  36.082  < 2e-16 ***
bloques     -0.1688717  0.0220336  -7.664  1.8e-14 ***
```

	DL 50	LI	LS
Bloque 1	82.747	63.454	106.559
Bloque 2	103.434	77.831	133.472
Bloque 3	124.121	92.208	160.385

Isolate Patient 57

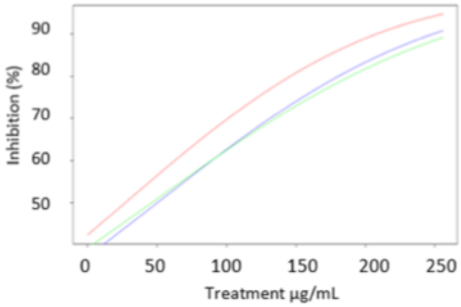


```
Coefficients:
             Estimate Std. Error z value Pr(>|z|)
(Intercept) -0.0860555  0.0575734  -1.495  0.13499
Treatment    0.0065633  0.0003229  20.324  < 2e-16 ***
Replication -0.0885944  0.0272656  -3.249  0.00116 **
```

	DL 50	LI	LS
Replication	25.858	1.119	57.268
Replication	41.373	6.006	81.108
Replication	54.303	10.892	104.948

Isolate Patient 40

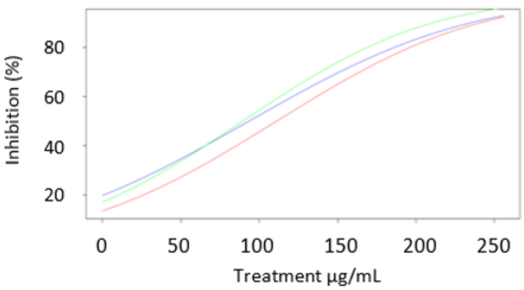


```
Coefficients:
             Estimate Std. Error z value Pr(>|z|)
(Intercept) -1.1478090  0.0563882 -20.355  < 2e-16 ***
Treatment    0.0096620  0.0002657  36.369  < 2e-16 ***
Replication  0.1053848  0.0254454   4.142 3.45e-05 ***
```

	DL 50	LI	LS
Replication	108.606	86.657	131.418
Replication	98.262	71.342	125.399
Replication	85.333	56.027	119.381

Isolate Patient 36

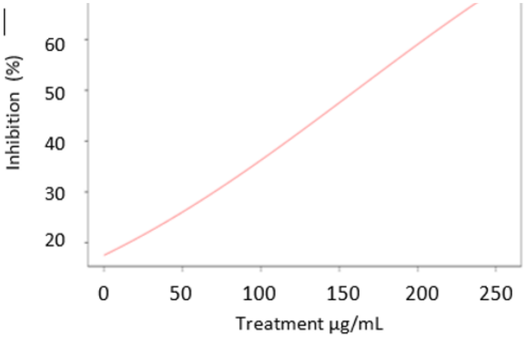


```
Coefficients:
             Estimate Std. Error z value Pr(>|z|)
(Intercept) -0.9452154  0.0396696  -23.83   <2e-16 ***
Treatment    0.0058000  0.0001572   36.91   <2e-16 ***
Replication  0.0072691  0.0169221    0.43    0.668
```

	DL 50	LI	LS
Replication	160.323	145.951	176.511

Isolate Patient 23

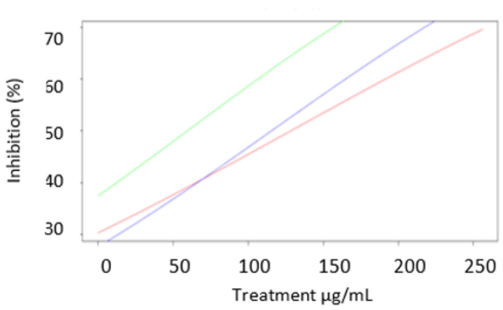

Coefficients:

	Estimate	Std. Error	z value	Pr(>|z|)	
(Intercept)	-0.8048699	0.0438094	-18.372	<2e-16	***
Treatment	0.0048875	0.0001788	27.338	<2e-16	***
Replication	0.1621786	0.0179765	9.022	<2e-16	***

	DL 50	LI	LS
Replication	131.878	99.693	168.145
Replication	98.262	61.986	140.181
Replication	64.646	24.279	112.216

Isolate Patient 60

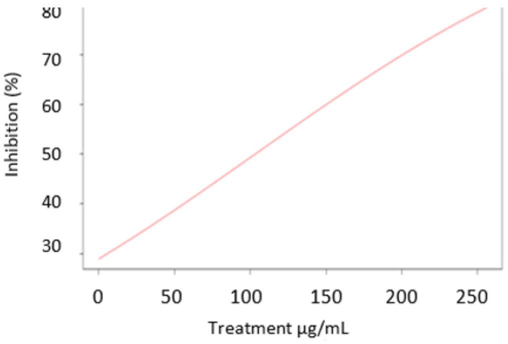

Coefficients:

	Estimate	Std. Error	z value	Pr(>|z|)	
(Intercept)	-0.5482059	0.0549694	-9.973	<2e-16	***
Treatment	0.0053810	0.0002354	22.862	<2e-16	***
Replication	-0.0035630	0.0237259	-0.150	0.881	

	DL 50	LI	LS
Replication	103.434	85.975	123.624

Isolate Patient 41

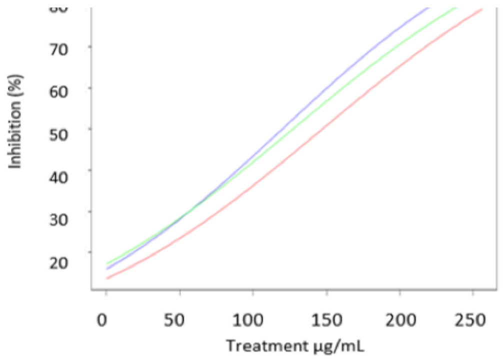

Coefficients:

	Estimate	Std. Error	z value	Pr(>|z|)	
(Intercept)	-1.1632422	0.0466028	-24.961	< 2e-16	***
Treatment	0.0076343	0.0002067	36.942	< 2e-16	***
Replication	0.0755058	0.0195855	3.855	0.000116	***

	DL 50	LI	LS
Replication	142.222	119.217	168.335
Replication	131.878	105.047	163.199
Replication	121.535	90.876	158.062

Isolate Patient 42

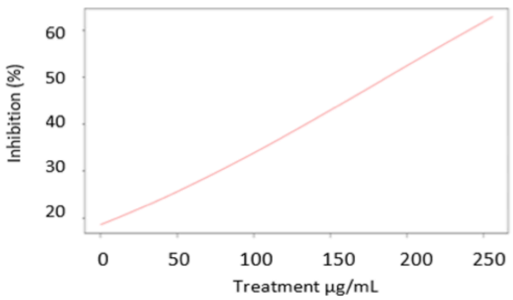

Coefficients:

	Estimate	Std. Error	z value	Pr(>|z|)	
(Intercept)	-0.9316093	0.0417345	-22.322	<2e-16	**
Treatment	0.0047669	0.0001477	32.266	<2e-16	**
Replication	0.0178563	0.0170924	1.045	0.296	

	DL 50	LI	LS
Replication	186.182	169.161	207.799

Control Group2

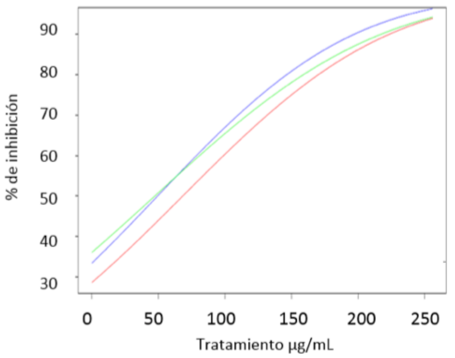


```
Coefficients:
             Estimate Std. Error z value Pr(>|z|)
(Intercept) -0.597133   0.031038 -19.239  < 2e-16 ***
tratamiento  0.008109   0.000158  51.307  < 2e-16 ***
bloques      0.075481   0.012979   5.815 6.05e-09 ***
```

	DL 50	LI	LS
Bloque 1	64.646	51.784	77.815
Bloque 2	54.303	39.770	71.418
Bloque 3	46.545	27.757	65.021

Isolate Patient 44

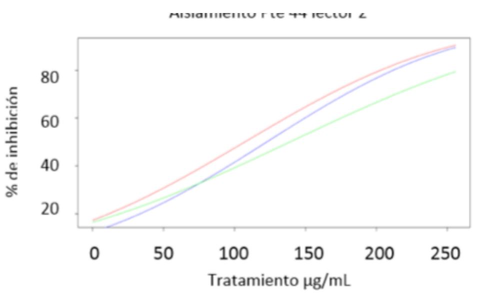


```
Coefficients:
              Estimate Std. Error z value Pr(>|z|)
(Intercept) -0.8320812  0.0321049 -25.918  < 2e-16 ***
tratamiento  0.0083683  0.0001473  56.806  < 2e-16 ***
bloques     -0.0949337  0.0142564  -6.659 2.76e-11 ***
```

	DL 50	LI	LS
Bloque 1	111.191	96.647	125.898
Bloque 2	121.535	104.406	141.078
Bloque 3	134.464	112.165	156.259

Isolate Patient 48

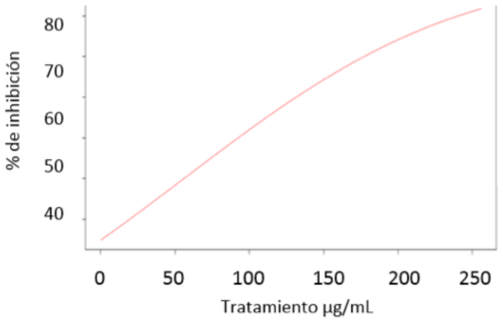


```
Coefficients:
              Estimate Std. Error z value Pr(>|z|)
(Intercept) -0.4175882  0.0371393  -11.24   <2e-16 ***
tratamiento  0.0069462  0.0001841   37.73   <2e-16 ***
bloques      0.0139521  0.0156811    0.89    0.374
```

	DL 50	LI	LS
Bloque 1	56.888	64.379	48.361

Isolate Patient 50

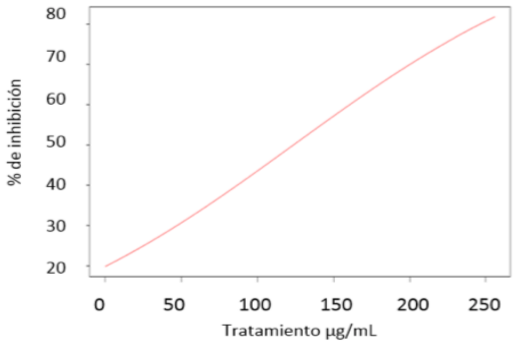


```
Coefficients:
              Estimate Std. Error z value Pr(>|z|)
(Intercept) -0.7916456  0.0392656 -20.161   <2e-16 ***
tratamiento  0.0068593  0.0001707  40.181   <2e-16 ***
bloques     -0.0276865  0.0168688  -1.641    0.101
```

	DL 50	LI	LS
Bloque 1	124.121	112.348	135.858

Isolate Patient 26

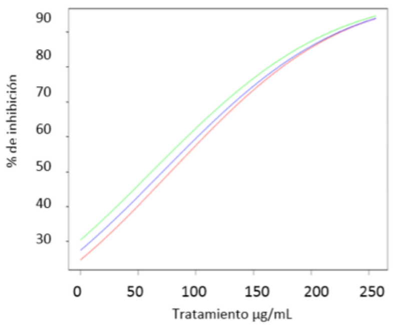


```
Coefficients:
             Estimate Std. Error z value Pr(>|z|)
(Intercept) -0.7358515  0.0328646 -22.390  < 2e-16 ***
tratamiento  0.0084262  0.0001552  54.303  < 2e-16 ***
bloques      0.0706086  0.0137278   5.143  2.7e-07 ***
```

	DL 50	LI	LS
Bloque 1	80.161	65.803	93.028
Bloque 2	69.818	54.610	87.665
Bloque 3	62.060	43.417	82.302

Isolate Patient 61

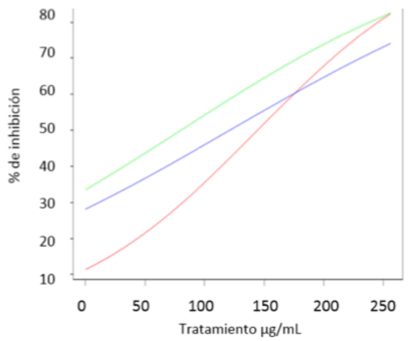


```
Coefficients:
              Estimate Std. Error z value Pr(>|z|)
(Intercept) -1.2207434  0.0801513 -15.230  < 2e-16 ***
tratamiento  0.0060390  0.0003283  18.397  < 2e-16 ***
bloques      0.2502107  0.0327201   7.647 2.06e-14 ***
```

	DL 50	LI	LS
Bloque 1	160.323	112.082	220.810
Bloque 2	118.949	64.933	186.436
Bloque 3	77.575	17.783	152.062

Isolate Patient 63

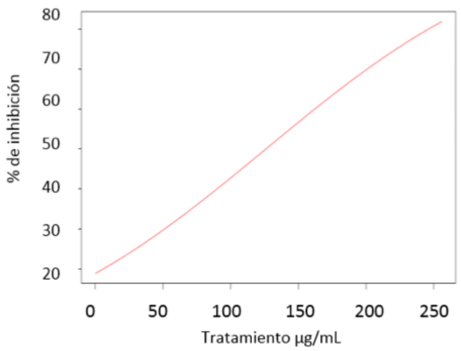


```
Coefficients:
             Estimate Std. Error z value Pr(>|z|)
(Intercept) -2.08564    0.07403 -28.173   <2e-16 ***
tratamiento  0.46592    0.01309  35.596   <2e-16 ***
bloques      0.04674    0.02502   1.868   0.0618
```

	DL 50	LI	LS
Bloque 1	126.707	110.989	143.105

Control Group3

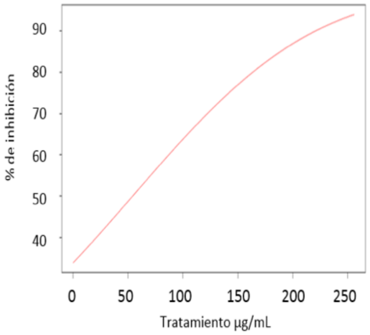


```
Coefficients:
             Estimate Std. Error z value Pr(>|z|)
(Intercept) -0.3443794  0.0716944  -4.803 1.56e-06 ***
tratamiento  0.0077024  0.0003838  20.067  < 2e-16 ***
bloques     -0.0376051  0.0319305  -1.178    0.239
```

	DL 50	LI	LS
Bloque 1	54.303	41.104	69.941

Isolate Patient 43

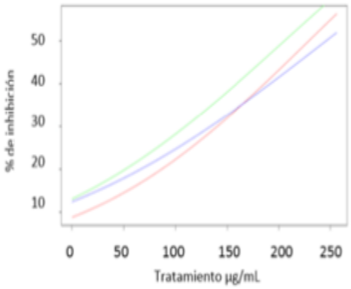


```
Coefficients:
             Estimate Std. Error z value Pr(>|z|)
(Intercept) -1.3858674  0.0418221 -33.137  < 2e-16 ***
tratamiento  0.0052959  0.0001529  34.639  < 2e-16 ***
bloques      0.0910909  0.0175906   5.178 2.24e-07 ***
```

	DL 50	LI	LS
Bloque 1	245.656	210.459	282.696
Bloque 2	227.555	188.025	271.358
Bloque 3	209.454	165.590	260.020
